# Enhancing the health of NHS staff: eTHOS — protocol for a randomised controlled pilot trial of an employee health screening clinic for NHS staff to reduce absenteeism and presenteeism, compared with usual care

**DOI:** 10.1186/s40814-022-01095-z

**Published:** 2022-07-27

**Authors:** Rachel Adams, Rachel Jordan, Peymané Adab, Tim Barrett, Sheriden Bevan, Lucy Cooper, Ingrid DuRand, Pollyanna Hardy, Nicola Heneghan, Kate Jolly, Sue Jowett, Tom Marshall, Margaret O’Hara, Kiran Rai, Hugh Rickards, Ruth Riley, Steven Sadhra, Sarah Tearne, Gareth Walters, Elizabeth Sapey

**Affiliations:** 1 Institute of Applied Health Research, University of Birmingham, Birmingham, B15 2TT UK; 2 Birmingham Children’s Hospital, Steelhouse Lane, Birmingham, B4 6NH UK; 3 Hereford County Hospital, Stonebow Road, Hereford, HR1 2ER UK; 4 National Perinatal Epidemiology Unit Clinical Trials Unit, University of Oxford, Oxford, UK; 5 School of Sport, Exercise and Rehabilitation, University of Birmingham, Birmingham, B15 2TT UK; 6 Public and Patient Involvement and Engagement, University Hospitals Birmingham NHS Foundation Trust, Birmingham, B15 2TH UK; 7 Institute of Clinical Sciences, University of Birmingham, Birmingham, B15 2TT UK; 8 National Centre for Mental Health, Barberry Building, 25 Vincent Drive, Birmingham, B15 2FG UK; 9 Birmingham Heartlands Hospital, Bordesley Green East, Birmingham, B9 5SS UK; 10 Institute of Inflammation and Ageing, University of Birmingham, Birmingham, B15 2TT UK; 11 Respiratory Medicine and General Internal Medicine, University Hospitals Birmingham NHS Foundation Trust, Queen Elizabeth Hospital Birmingham, Birmingham, B15 2TH UK

**Keywords:** Healthcare workers, Absenteeism, Presenteeism, Occupational health, Health screening, Employee health, NHS, Randomised controlled pilot trial

## Abstract

**Background:**

Staff absenteeism and presenteeism incur high costs to the NHS and are associated with adverse health outcomes. The main causes are musculoskeletal complaints and mental ill-health, which are potentially modifiable, and cardiovascular risk factors are also common. We will test the feasibility of an RCT to evaluate the clinical and cost-effectiveness of an employee health screening clinic on reducing sickness absenteeism and presenteeism.

**Methods:**

This is an individually randomised controlled pilot trial aiming to recruit 480 participants. All previously unscreened employees from four hospitals within three UK NHS hospital Trusts will be eligible. Those randomised to the intervention arm will be invited to attend an employee health screening clinic consisting of a screening assessment for musculoskeletal (STarT MSK and STarT Back), mental (PHQ-9 and GAD-7) and cardiovascular (NHS Health Check if aged ≥ 40, lifestyle check if < 40 years) health. Screen positives will be given advice and/or referral to recommended services. Those randomised to the control arm will receive usual care. Participants will complete a questionnaire at baseline and 26 weeks; anonymised absenteeism and staff demographics will also be collected from personnel records. The co-primary outcomes are as follows: recruitment, referrals and uptake of recommended services in the intervention arm. Secondary outcomes include the following: results of screening assessments, uptake of individual referrals, reported changes in health behaviours, acceptability and feasibility of intervention, indication of contamination and costs. Outcomes related to the definitive trial include self-reported and employee records of absenteeism with reasons. Process evaluation to inform a future trial includes interviews with participants, intervention delivery staff and service providers receiving referrals. Analyses will include presentation of descriptive statistics, framework analysis for qualitative data and costs and consequences presented for health economics.

**Discussion:**

The study will provide data to inform the design of a definitive RCT which aims to find an effective and cost-effective method of reducing absenteeism and presenteeism amongst NHS staff. The feasibility study will test trial procedures, and process outcomes, including the success of strategies for including underserved groups, and provide information and data to help inform the design and sample size for a definitive trial.

**Trial registration:**

ISRCTN reference number 10237475.

## Introduction

The National Health Service (NHS) in the UK is one of the top ten employers in the world, with 1.3 m employees [1]. However, sickness absenteeism costs the NHS approximately £2.4 billion per year [2], with an annual average of just under 10 absence days per employee [3]. In 2016, this was 46% higher than other UK industries and 27% higher than the average in the public sector [4, 5] and is associated with worse patient outcomes [2, 6–10]. For example, there is an established link between staff mental illness and poor patient outcomes such as patient satisfaction and medication errors [11–13]. A greater potential cost is that of presenteeism (attending work whilst unwell) [14–16], with 56% of NHS staff in 2009 reporting pressure to attend work when feeling unwell [17]. Workers often cycle between absenteeism and presenteeism.

The main cause of sickness absenteeism in the NHS is mental ill-health at 25.4% of all recorded days lost in 2019, nearly double that of 2010 [18] and peaking at 32.4% during the first wave of the COVID-19 pandemic [19]. This is followed by musculoskeletal (MSK) complaints affecting 16% [20]. Poor lifestyle (smoking, lack of physical activity) and overweight/obesity are also important independent determinants of absenteeism [21–25] and presenteeism [26–28], with cardiovascular disease being up to five times greater amongst staff over 50 years old (unpublished local NHS data) [29]. Absenteeism varies by occupational group, being highest in the lowest paid (healthcare assistants), professions allied to medicine and infrastructure support staff [30]. Presenteeism rates follow similar patterns [31]. Since the pandemic, rates of absenteeism have risen, and reports of mental ill-health have increased [32].

In response to the high levels of staff ill-health and absenteeism, NHS England created a “Healthy Workforce Programme”, supported by the Royal College of Physicians [33], with a £450 m financial incentive for Trusts to improve staff health and well-being and thereby patient care [34]. Key actions included making the NHS cardiovascular disease (CVD) health check (for those aged 40–74 years without pre-existing diabetes or CVD) [35] more accessible for staff in the workplace and improving access to physiotherapy, mental health, weight management and smoking cessation services [36]. In most hospitals, occupational health services, which are often outsourced, do not have a preventive or well-being role, and therefore, new initiatives are required.

There are several systematic reviews and many individual studies which evaluate the effectiveness of workplace health promotion or return-to-work programmes [27, 37–39], but few are conducted amongst healthcare staff, and they rarely consider impact on absenteeism or presenteeism or include a control group. None has evaluated the cost-effectiveness of interventions targeted at screening for and early management of the main causes of absenteeism in healthcare settings — essential evidence before Trusts will invest limited healthcare resources. The evidence available reinforces the need to focus on high-risk groups, provide interventions of sufficient intensity and optimise attendance to maximise the chances of interventions being effective [40, 41]. The most effective model is likely to be a combination of health screening and health/wellness programmes with targeted interventions [42]. Based on learning from a previous pilot self-referral employee health screening clinic set up at the Queen Elizabeth Hospital Birmingham (QEHB), we have developed a health screening service for hospital employees, including pathways for direct invitation, assessment for CVD, MSK and mental health problems (the three major causes of staff absenteeism) and onward referral. The pilot clinic demonstrated that 41% of attendees were overweight, 18% obese, 27% inactive, 12% were smokers, 11% had hypertension and 11% high cholesterol, 13% had moderate and 10% severe anxiety and 8% had moderate and 4% severe depression. Overall, around a third required at least one onward referral (unpublished data). However, the clinic had poor engagement from lower paid staff, ethnic minorities and night shift workers, and therefore, a more inclusive approach to promotion of the service is required to be fully effective. We present the protocol of a pilot randomised controlled trial (RCT) to assess the feasibility of conducting a full RCT to evaluate this service in reducing absenteeism and presenteeism, as well as to evaluate its cost-effectiveness.

## Methods

### Aims and objectives

The aim of this pilot trial is to test the feasibility of a definitive RCT evaluating the clinical and cost-effectiveness of a complex intervention (health screening clinic), compared to usual care, in reducing absenteeism and presenteeism amongst NHS staff.

Objectives for the feasibility study are as follows:

- Describe recruitment rates.
- Describe participant characteristics and assess generalisability compared to the hospital workforce.
- Describe screening assessment results.
- Describe recommended referrals and their uptake.
- Assess the feasibility of measuring the primary and secondary outcomes and obtain information to inform the sample size for a full RCT.
- Assess levels of contamination between intervention and usual care arms.
- Describe and explain the fidelity to the intervention and evaluate views, experiences and acceptability of participants and intervention delivery staff.
- Explore the feasibility of such a service in other NHS and external settings, e.g. ambulance service, GP (general practitioner) practices and commercial organisations, explored in the process evaluation.
- Quantify the costs of undertaking the screening service and its consequences.

### Design and setting

A multicentre, parallel group, open, individually randomised pilot RCT of a complex intervention comparing an employee health screening clinic with usual care in four NHS Hospital Trusts in the West Midlands, including three large urban hospitals and one rural district general hospital (Table 1). This provides good generalisability and allows testing practicality in a range of sites. On balance, we have chosen an individually randomised trial as we feel this is more feasible, requiring fewer participants and fewer hospitals. We anticipate risk of contamination to be low as the full screening assessment, and tailored individual recommendations will only be accessible by invite and subsequent participation in the intervention arm of the trial. However, this will be assessed in the trial to decide whether a cluster trial is warranted instead.

**Table 1:** Description of study sites

Hospital	Staff	Setting
Queen Elizabeth Hospital Birmingham (QEHB) (University Hospitals Birmingham (UHB) NHS Foundation Trust)	9000	The largest single site hospital in the country. Regional centre for cancer, largest solid organ transplantation programme in Europe, a regional Neuroscience and Major Trauma Centre and includes The Royal Centre for Defence Medicine.
Heartlands hospital (University Hospitals Birmingham NHS Foundation Trust)	11000	Includes four secondary care city-based hospital sites (Heartlands, Good Hope, Solihull and the Chest Clinic), one of the top five employers in the West Midlands. We will focus on the Heartlands site
Birmingham Children’s Hospital (Birmingham Women’s and Children’s NHS Foundation Trust)	6000	Secondary and tertiary care hospital serving 384,000 women and children annually
Hereford hospital (Wye Valley NHS Trust)	3000	One of the smallest rural district general hospitals in England, serving a population of 180,000

### Participants, eligibility, recruitment and consent

All employees in the participating hospitals who are able to provide informed consent will be eligible to participate except those who have previously attended a pilot clinic at QEHB or who are currently taking part in another intervention trial.

In order to be accessible to the full workforce and promote equitable recruitment of participants on gender, race, staff group and staff grade, participants will be invited through multiple approaches including email, information on payslips, mail outs, personal invitation, staff meetings, noticeboards and ward champions, in several phases, designed as far as possible to reflect the workforce characteristics of each hospital. Departmental recruitment information will be collected, so we can analyse how the trial was promoted across the Trusts. Reminder letters will be sent to nonresponders after 2 weeks and a further two if necessary. Posters advertising the trial will be displayed in departments/wards for a minimum of 2 weeks (or more if local arrangements require) before invitations are sent out during which time staff can request not to receive an invitation. Local staff will be engaged to raise trial awareness and arrange cover to allow attendance.

Consent will be obtained electronically online, on participants’ own electronic devices or with the help of trial staff at the health screening clinic. The participant information sheet will be available on the study website and in paper format; participants may contact the research team by telephone or request contact from the research team via the trial website to ask any questions. Consent will be confirmed at each follow-up appointment.

### Data collection and management

Table 2 shows the data collection schedule for participants. Study data will be entered into a REDCap1^1^ (Research Electronic Data Capture) database custom designed and hosted by the University of Birmingham either directly by participants or the clinic staff [43, 44]. Participants will complete the baseline questionnaire at a time convenient to them. Prompts will be sent after 24 h, 2 days and 7 days where the baseline questionnaire remains incomplete.

**Table 2:** Schedule of assessments

Visit	Eligibility screening, consent and randomisation	Baseline	Intervention screening visit	26-week follow-up data collection	Between baseline and 26 weeks
Timeframe for follow-up data collection				+/−4 weeks	
Trial registration	x				
Consent to eligibility screening	x				
Eligibility screening	x				
Participant information and contact details	x				
Valid informed consent	x				
Staff payroll number	x				
NHS number (optional)	x				
Demographics (DOB, gender, education, marital status)		x			
Smoking status		x		x	
Exercise level (GPPAQ questionnaire)		x	x	x	
Ethnicity		x			
Diagnosed medical conditions		x			
Current medications		x			
Health status (EQ-5D questionnaire)		x		x	
Height		x			
Weight		x		x	
Health service utilisation		x		x	
Current employment		x		X	
Absenteeism and presenteeism (WHO-HPQ questionnaire)		x		x	
Occupational health resource utilisation		x		x	
Randomisation	x				
**Health screening (intervention group only)**					
**Musculoskeletal health (if applicable)**					
STarT Back screening tool (participant completion)			x		
STarT Back screening tool (review)			x		
STarT MSK screening tool (participant completion)			x		
STarT MSK screening tool (review)			x		
OMPSQ tool (participant completion)			x		
OMPSQ tool (review)			x		
Impact on work questions: musculoskeletal health			x		
**Mental health (if applicable)**					
GAD7 questionnaire (participant completion)			x		
GAD7 questionnaire (review)			x		
PHQ9 questionnaire (participant completion)			x		
Impact on work questions; mental health			x		
**Cardiovascular health (if applicable)**					
Personal details checked from baseline (age, ethnicity)					
BMI			x		
Smoking status (review from baseline questionnaire)			x		
Alcohol intake (review of AUDIT C questionnaire)			x		
Exercise level (review of GPPAQ questionnaire)			x		
Cardiovascular risk calculator (QRISK2 score)			x		
**Clinical measures (if age > = 40 years)**					
ECG			x		
Pulse			x		
Blood pressure			Up to 3 readings		
Blood tests (criteria for taking blood must be met)			x		
Cholesterol			x		
HbA1C (if indicated as per NHS Health Check)			x		
eGFR (if indicated as per NHS Health Check) (calculated using creatinine or U&Es, according to Trust policy)			x		
Recommendations from the health screening clinic (intervention group only)				x	
Recently diagnosed health conditions				x	
Trust staff characteristics	x				
Trust absenteeism rates	x				
Participant absenteeism data	x			x	
**Process evaluation (if applicable)**					
Valid informed consent					x
Qualitative interview/focus group					x

Self-reported data will be collected on all participants prior to randomisation, but after consent, including the following: contact details, demography, employment details, selected diagnosed medical conditions and current medications, absenteeism (World Health Organisation Health and Work Performance Questionnaire — WHO-HPQ) [45], presenteeism (WHO-HPQ) [45], health-related quality of life (HRQoL EuroQol 5 level — EQ-5D 5L) [46], smoking status, height, weight, exercise levels (General Practice Physical Activity Questionnaire — GPPAQ) [47], receipt of NHS Health Check and health service utilisation. We will also collect NHS number where available (this is optional) to allow (and assess the feasibility of) future linkage to routine data such as hospital admissions, general practitioner (GP) records, other healthcare utilisation and mortality data. Consent will be obtained to collect the following data from human resources (HR) records:

- Participant age, sex, ethnicity, staff group and staff grade
- Number of hours contracted to work
- Hours worked — full-time equivalent
- Sickness absenteeism and non-sickness absences relating to COVID-19 only, for the previous 24 months and follow-up (start/end dates, duration in days, recorded reason)
- Leaving date (should participant leave employment of the Trust during trial participation)

At 26 weeks, participants will be sent a follow-up questionnaire for online completion to report uptake of any recommended services (intervention arm only) and other outcomes as detailed below. Linked personnel data will be obtained on all consented patients including absenteeism at 26-week follow-up to allow comparison pre/post intervention.

Additional anonymised data will be collected at the time of site set-up from hospital HR records, at whole hospital level and for those invited and on age, sex, ethnicity, job role, days and reasons for absenteeism, in order to assess the generalisability of the included participants.

Respondents will also be provided with the option to provide reasons for taking/not taking part in the study. This will be anonymised.

### Allocation to trial arm

On completion of the baseline questionnaire, clinic staff will receive an alert to check eligibility criteria and consent, and participants will then be randomised to intervention or control arm. Randomisation will be at the level of the individual in a 1:1 ratio, using a minimisation algorithm to ensure balance on the following variables: age, sex, self-reported job categories, nightshift work and centre. To avoid predictability, a “random element” will be included in the minimisation algorithm. Given the nature of the intervention, blinding will not be possible.

### Intervention

**Fig. 1:**
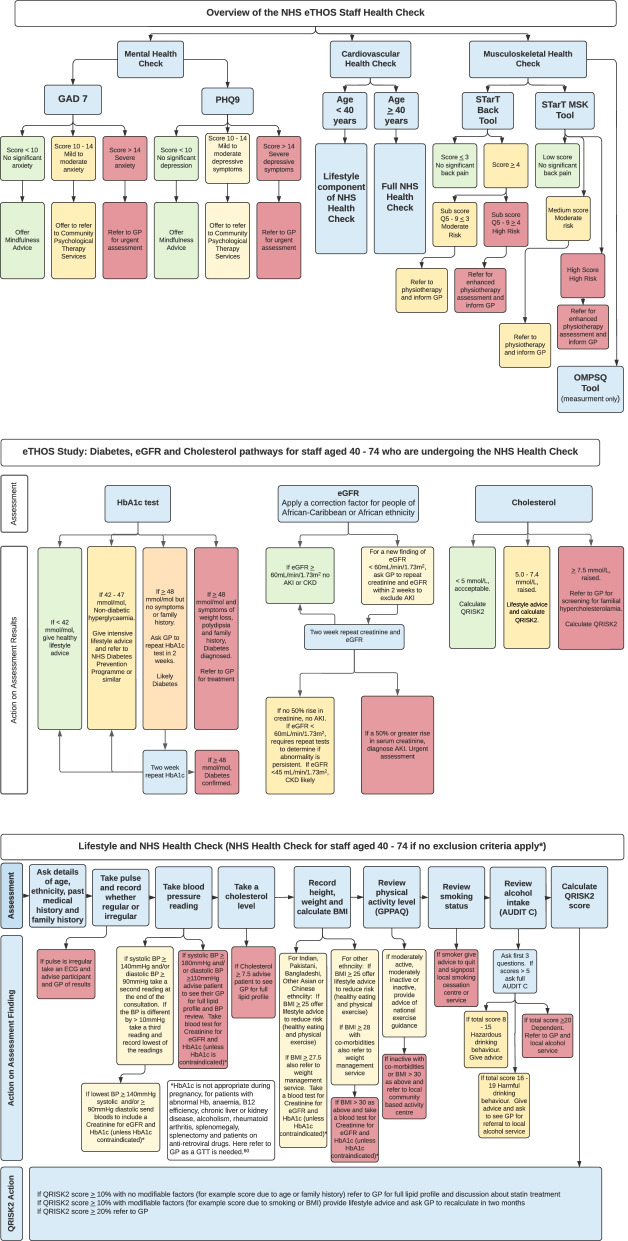
Overview of NHS staff health screening check

The intervention is an employee staff health screening clinic available during usual work time (9 am–5 pm) and some evenings and weekends [37], administered by trained clinic nurses. It will last approximately 1 h and consist of two stages: (1) screening assessment for musculoskeletal, mental and cardiovascular health (which also includes lifestyle components such as alcohol consumption assessment), followed by (2) appropriate advice and/or referral of screen positives to appropriate services for management as per NHS/NICE recommendations (see Fig. 1 for overview) and personalised care pathways. There are 3 components to the screening intervention.

#### Musculoskeletal health

We will use the Keele STarT back screening tool [48, 49] to categorise risk of future disability of low back pain and STarT MSK for non-back complaints [50, 51], and Orebro musculoskeletal pain screening questionnaire (short) — OMPSQ [52] — to enable comparison with international studies. Based on a model of stratified care, those with moderate scores on the STarT Back or STarT MSK tools will be referred to physiotherapy teams (according to local pathways), and those with high scores will receive enhanced physiotherapy including cognitive behavioural therapy to address associated psychological problems (Fig. 1).

#### Mental health

We will use the GAD7 [53, 54] (anxiety) and PHQ9 [54, 55] (depression) screening questionnaires to assess mental health. Participants without significant anxiety/depressive symptoms will be offered online mindfulness advice [56]. Those who are mildly/moderately affected will be advised to seek support from local counselling services such as Birmingham Healthy Minds [57] or, if severely affected, referred to their GP for immediate treatment (Fig. 1).

#### Cardiovascular health

Participants aged 40 years and over will receive the NHS Health Check [35]. This includes lifestyle checks (BMI (body mass index), exercise level (GPPAQ questionnaire [47]), smoking status and alcohol intake (alcohol use disorders identification test for consumption (AUDIT-C) questionnaire [58]), QRISK2 score [59]) and clinical measures (pulse, blood pressure, ECG, cholesterol, and HbA1C, eGFR and creatinine tests if appropriate). Actions will include referral to GP, weight management, smoking cessation and alcohol reduction services, as well as brief advice on exercise and diet according to UK recommendations (Fig. 1). The dementia awareness component for those over 65 will be excluded as there are few employees in this category. Participants aged under 40 years will be assessed for restricted lifestyle components of the NHS Health Check: BMI, exercise level, smoking status and alcohol intake, with the same advice applied and referrals to appropriate services. Blood samples will not be collected from participants aged under 40 years.

Participants reporting diagnosed conditions in any of the above 3 categories will not receive that element of the intervention. Results will be sent to participants’ GPs for their records if the participants provide consent.

#### Occupational health

All participants with an identified health condition will also be asked whether their condition is affected by/or affects their ability to work and what adjustments (if any) at work may improve their workability. If yes, they will be offered an optional referral to occupational health.

### Usual care

Participants in the control arm will receive usual access to medical services for management of any presenting condition (either through occupational health or their GP) and will remain eligible for the usual NHS Health Check via their GP.

### Outcomes

#### Primary outcomes and stop/go criteria

There are three co-primary outcomes:

- Recruitment (consented) as a proportion of those invited
- Direct referral to any recommended services as a result of the three screening components (usually GP, physiotherapy, community psychological services depending on local pathways at the time of the study) (intervention arm only)
- Attendance at any recommended services (self-report at 26 weeks, intervention arm only)

These will inform criteria to progress to the definitive trial (Table 3).

**Table 3:** Progression criteria informing decision about full trial

		Progression criteria		
	Description	Green (go)	Amber (pause)	Red (stop)
Recruitment^a^	% of invited employees consenting to take part	> 25%	15–25%	< 15%
Referred directly to a service^b^	% of participants randomised to intervention arm referred to specified services Measured at 26 weeks	> 30%	10–30%	< 10%
Attendance at referrals^c^*	% of referrals which resulted in self-reported attendance at the service at least once. Measured at 26 weeks	> 50%	30–50%	< 30%
**Action**		**If ALL criteria are GREEN, proceed to full trial with protocol unchanged**	**If ANY of these criteria are AMBER, adapt protocol appropriately using information from the process evaluation before proceeding to full trial**	**If ALL of these criteria are RED, consider whether current protocol is not feasible** **If ONE OR TWO of these criteria are RED, consider whether adaptations are needed**

^a^A recent study of low-paid government workers receiving an NHS Health Check in the workplace demonstrated benefits to cardiovascular health with 20% uptake of the intervention [60] and experience from a primary care COPD screening trial with > 35% uptake [61] and primary care cohort with > 25% uptake [62]. ^b^A feasibility study of a cardiovascular health check in an NHS hospital showed that 33% required follow-up with a GP or other health professionals [63]; experience from our pilot clinic with cardiovascular and mental health checks showed that 35% required further follow-up. ^c^The feasibility study mentioned above also showed that 54% of those referred to further services had attended within 5 weeks, and most of the remainder intended to do so [63]. *We will present both % of referred participants attending at least one of their referrals (at least once) and % of total referrals resulting in an attendance

#### Secondary outcomes

- Baseline characteristics of included participants and hospital population for comparison
- Description of the results of the intervention screening assessments
- Number and type of direct referrals to recommended services (intervention arm only)
- Attendance at each individual recommended service (self-report at 26 weeks; intervention arm only)
- Lifestyle relevant to screening intervention advice and referrals (self-report at 26 weeks compared with baseline):
  - Physical activity index measuring exercise levels (GPPAQ) [47]
  - Smoking status
  - Weight (kg)
- Acceptability of intervention to participants and health screening clinic staff (interviews)
- Feasibility of trial processes (completeness of relevant data items, interviews)
- Indication of contamination (comparing pre/post data for health behaviours and healthcare/other service utilisation in control arm)

#### Outcomes related to the definitive trial

- Absenteeism at 26 weeks with reasons, measured by days and spells, is as follows:
  - Self-report absolute absenteeism, relative absenteeism and relative hours of work — for the last 7 days and last 28 days (WHO-HPQ) [45] at 26 weeks
  - Self-report absenteeism 6-month recall period at 26 weeks
  - Employee records of absenteeism at 26 weeks, which will be the primary outcome of the definitive trial, using routine collected data from the NHS Electronic Staff Record Programme, linked to employee ID and provided directly by electronic HR records
- Presenteeism at 26 weeks (self-report absolute presenteeism and relative presenteeism for the last 28 days (WHO-HPQ)) [45]
- Attendance at occupational health service (self-report at 26 weeks)
- Healthcare utilisation (self-report at 26 weeks (e.g. GP consultations, hospital admissions))
- EQ-5D-5L index value measuring health-related quality of life (EuroQol EQ-5D 5-level) [46] at 26 weeks
- Patient (self-report questionnaire) and trial intervention resource use/costs
- Screening assessment duration and resources used

### Process evaluation

A mixed-methods process evaluation will explore programme reach; fidelity of screening delivery; attendance at referrals; participants’ views of the intervention; views and experiences of the training received by those delivering the intervention, its acceptability and satisfaction with the intervention overall; and views of providers of follow-on services, e.g. GPs and “healthy minds”. In addition, we will explore the views of potential beneficiaries, e.g. other NHS organisations who might be willing to run the trial such as ambulance services and GP groups and private, non-healthcare organisations who might be interested in delivering and evaluating such a service for their setting.

Quantitative data to support the process evaluation will be obtained from the following:

- *Recruitment and follow-up data*, e.g. response rate; proportions of those invited consenting, being randomised and attending the screening; follow-up at 26 weeks
- *Baseline questionnaire* to assess characteristics, e.g. data on age, ethnicity, marital status, Index of Multiple Deprivation, educational attainment and employment role of the employees recruited to study (programme reach)
- *Logs* kept by the staff health screening programme recording attendance, screening tests undertaken and duration of contacts (fidelity)
- *Healthcare issues* identified at the employee screening and referrals made to GP and other services (fidelity)
- *Self-report of attendance at recommended services* from follow-up questionnaires (uptake)
- *Brief questionnaire to staff* not taking up the offer of the study to ascertain reasons for not participating and a question to participants to ascertain which recruitment method most prompted them to participate (reach)

Focused qualitative interviews lasting approximately 30 min either face to face, by voice over Internet protocol (e.g. zoom) or telephone according to participant preference will be conducted with the following:

- Up to 40 participants, sampled on age, gender and ethnicity, occupation, site, shift pattern and department, selected from those randomised.

Those in the intervention group are as follows:

A. Did not attend screening
B. Did not need referral to other services
C. Needed referral to other services

We will explore acceptability, barriers and facilitators to the intervention, views on the benefits of the intervention, their experience of it, its value, disbenefits, fears about confidentiality of workplace service, views on occupational health services and their experience of being referred to other services.

D. The usual care group﻿: to explore awareness of the trial and its intervention and if prompted to seek a health check outside of the trial

- Staff undertaking health checks: exploring the feasibility of implementing the intervention in theory/practice, training and their experiences of delivery in relation to the different cadres of staff
- Up to 12 GPs and staff delivering relevant services exploring their experience of receiving referrals, the acceptability of the process and availability of appropriate referral pathways from primary care, e.g. smoking cessation and physiotherapy services
- Up to 10 potential beneficiaries (e.g. ambulance service or private sector companies) exploring the feasibility and acceptability of conducting the trial and/or delivering the intervention in other contexts.

### Sample size

The study is not designed or powered to detect a statistically significant difference in efficacy between the two trial arms. We aim to recruit 480 participants (20 per week) in 24 weeks. With this sample size, the 95% confidence interval (CI) for the proportion of staff recruited can be estimated to be 4% either side of the estimate (e.g. for a 25% recruitment rate, the 95% CI will lie between 21 and 29%).

### Analyses

#### Statistical methods

The primary comparison groups will be composed of those randomised to the health screening clinic arm (intervention) and those randomised to the usual care arm (control). All analyses will use the intention to treat principle, i.e. participants will be analysed in the treatment group to which they were randomised, irrespective of adherence or other protocol deviation.

Analyses of feasibility, absenteeism, presenteeism and clinical outcomes will take the form of simple descriptive statistics (e.g. proportions and percentages, means and standard deviations). The primary outcomes will be presented descriptively as proportions with 95% confidence intervals.

Secondary outcomes will be presented descriptively as proportions for categorical data and means and standard deviations/medians and interquartile ranges for continuous data, along with 95% confidence intervals. No formal statistical analysis will be undertaken.

Due to the electronic system of collecting data, it is anticipated that missing data will be minimal. The main analysis will use available data online; however, the amount of missing data will be assessed in order to inform decision regarding data collection for the definitive trial.

#### Qualitative analysis

Interviews and focus groups will be audio-recorded and transcribed clean verbatim for analysis. A thematic analysis of content will be informed by the Framework analytical approach [64]. Analysis and discussion will include the experienced qualitative team in partnership with the patient and public involvement (PPI) group to provide multiple perspectives on the data. Data collection and analysis will run concurrently so that emergent analytical themes can inform further data collection.

### Health economic analysi**s**

To inform the design of a full economic evaluation, we will conduct a descriptive cost-consequence analysis presenting disaggregated information on all relevant resource use and outcomes for both trial arms and costs of screening. Resource use, absenteeism, productivity loss data and the completeness of the data will be assessed. Resource use required for screening will be multiplied by unit costs obtained from standard sources (NHS Reference Costs, Unit Costs of Health and Social Care) and healthcare providers. Responses to the EQ-5D 5L questionnaire, valued using the crosswalk algorithm, will be used to calculate quality-adjusted life years (QALYs) using the area under the curve method. Analyses will be mainly descriptive, and all costs and outcomes will be summarised using means and 95% confidence intervals.

### Current status of trial and impact of COVID-19 pandemic

Current status is as follows: At submission this trial was still under recruitment. Ethical approval for the trial was obtained in March 2020, but was not able to proceed due to the COVID-19 pandemic. Two hospital sites granted permission to commence the trial in December 2020, but after a very short period, recruitment was paused again due to a spike in COVID-19 cases. The trial recommenced in May 2021 in all four sites. A 6-month extension to the trial was been awarded from the funder until October 2021.

### Patient and public involvement (PPI)

The study design has been informed by a survey of 60 QEHB staff and meetings with patients, relatives, friends accompanying them, members of the public, UHB’s hospital executive board, senior/middle managers, local GPs, the University of Birmingham’s patient advisory group, staff consultations relating to a self-referral staff health screening clinic at QEHB, data from the annual NHS staff survey at QEHB (*n* = 3381 (35%) response) and a PPI group specifically set up to inform eTHOS throughout the trial, comprising 4–6 staff members and patients.

### Data storage and confidentiality

Participants’ personal data will be held securely and treated as strictly confidential, in line with the Data Protection Act 2018. Electronic records will be held on a secure, password-protected, web-enabled customised database hosted by the Birmingham Clinical Trials Unit (BCTU). Paper records will be transferred from the participating study centres to the trial office at BCTU and kept in a locked cabinet in a locked room; participants will be asked to consent to this prior to entry into the trial. Any data processed outside BCTU will be anonymised. Data will be stored for 10 years following study completion.

### Monitoring

BCTU is the coordinating centre, registered with the UKCRC and has been involved in the development and design of the protocol since conception. A trial management group will oversee research methodology, clinical trial coordination, data management, statistical analysis, compliance with Good Clinical Practice and all regulatory requirements, including adverse event reporting, in conjunction with the sponsor.

A trial oversight committee (combining the function of trial steering and data monitoring committees) with an independent chair and lay representative will oversee, advise on and monitor the trial.

### Adverse events

No adverse or serious adverse events are expected for this trial; therefore, no adverse events data will be collected. Hospitalisations and deaths will be recorded as part of the trial data set.

### Protocol version

This publication is based on eTHOS protocol version 5.0 25 Oct 2021.

## Discussion

This important study is aimed at improving the well-being of healthcare staff, patient care and the economy and is the first of its kind to be conducted in the UK. It is a pilot trial in four NHS hospitals testing the feasibility of a large-scale RCT evaluating the clinical and cost-effectiveness of a hospital-based employee health screening clinic incorporating screening for musculoskeletal, mental and cardiovascular health with appropriate referrals in reducing absenteeism and presenteeism, in comparison with usual care. Processes have been put in place in order to ensure that those who find services harder to access will have the opportunity to take part in the study, and so that the trial will benefit those who most need it. The COVID-19 pandemic has disproportionately impacted the health of healthcare workers and highlighted the need for effective and cost-effective services to improve their health and well-being.

## Data Availability

The datasets will be available from the authors on reasonable request.

## Abbreviations

BCTU: Birmingham Clinical Trials Unit
BMI: Body mass index
CI: Confidence interval
CVD: Cardiovascular disease
GAD: Generalised Anxiety Disorder Questionnaire
GP: General practitioner
GPPAQ: General Practice Physical Activity Questionnaire
HR: Human resources
HRQoL: Health-Related Quality of Life
NHS: National Health Service
PPI: Patient and public involvement
QEHB: Queen Elizabeth Hospital Birmingham
RCT: Randomised controlled trial
UHB: University Hospitals Birmingham
WHO-HPQ: World Health Organisation Health and Work Performance Questionnaire

## Footnotes

1 A secure, web-based software platform designed to support data capture for research studies, providing (1) an intuitive interface for validated data capture, (2) audit trails for tracking data manipulation and export procedures, (3) automated export procedures for seamless data downloads to common statistical packages, and (4) procedures for data integration and interoperability with external sources
